# Supplementary material for: Lithium intercalated FeSe as a high-temperature superconducting ferromagnet
Source: Nat Commun. 2025 Aug 7;16:7305. doi: 10.1038/s41467-025-62624-x (PMC12331997; doi:10.1038/s41467-025-62624-x)
Supplement: Supplementary file 1 — Supplementary Information [file 41467_2025_62624_MOESM1_ESM.pdf]

Supplementary Information:

Lithium intercalated FeSe as a high-temperature superconducting ferromagnet

Yi Hu[1#], Keyi Liang[2#], Jie Li[3#], Zhijie Li[2], Fanyu Meng[4,5], Hechang Lei[4,5], Jiyuan Wang[1], Huizhen Wen[1], Ruozhou Zhang[2], Jiaqiang Cai[6], Jinglei Zhang[6], Yi Lu[3,7]*, Yihua Wang[2,8]*, Qi-Kun Xue[1,9,10]* and Ding Zhang[1,9,11,12]*

[1] State Key Laboratory of Low Dimensional Quantum Physics and Department of Physics, Tsinghua University, Beijing 100084, China

[2] State Key Laboratory of Surface Physics and Department of Physics, Fudan University, Shanghai 200433, China

[3] National Laboratory of Solid State Microstructures and Department of Physics, Nanjing University, Nanjing 210093, China

[4] School of Physics and Beijing Key Laboratory of Optoelectronic Functional Materials & MicroNano Devices, Renmin University of China, Beijing 100872, China

[5]Key Laboratory of Quantum State Construction and Manipulation (Ministry of Education), Renmin University of China, Beijing 100872, China

[6] Anhui Province Key Laboratory of Condensed Matter Physics at Extreme Conditions, High Magnetic Field Laboratory, HFIPS, Chinese Academy of Sciences, Hefei, Anhui 230031, China

[7] Collaborative Innovation Center of Advanced Microstructures, Nanjing University, Nanjing 210093, China

[8] Shanghai Research Center for Quantum Sciences, Shanghai 201315, China

[9] Beijing Academy of Quantum Information Sciences, Beijing 100193, China

[10] Southern University of Science and Technology, Shenzhen 518055, China

[11] Hefei National Laboratory, Hefei 230088, China

[12] RIKEN Center for Emergent Matter Science (CEMS), Wako, Saitama 351-0198, Japan

Contents

Supplementary Note 1 Extended DFT Results and Computational Details (Supplementary Fig. 1-2, Supplementary Table 1)

Supplementary Note 2 Characterization of pristine FeSe (Supplementary Fig. 3)

Supplementary Note 3 Extended data of sample S1 (Supplementary Fig. 4-6)

Supplementary Note 4 Extended data of sample S2 (Supplementary Fig. 7-13)

Supplementary Note 5 Broadening of superconducting transition (Supplementary Fig. 14)

Supplementary Note 6 Magnetometry after de-intercalation (Supplementary Fig. 15)

Supplementary Note 7 Extended data of samples S5 (Supplementary Fig. 16)

Supplementary Note 1 Extended DFT results and computational details

Monolayer and bulk Li_xFeSe were modeled using 2 × 2 × 1 and 2 × 2 × 2 supercells, respectively. Li dopants were positioned at hollow sites directly above or below Se ions, aligning with potential energy minima identified for Li intercalation in FeSe [1]. Structural configurations for different doping levels are illustrated in Supplementary Fig. 1. Symmetrically distinct doping sites and configurations were tested for the case of effective Hubbard U_{eff} =0 but were generally found to have higher total energies.

The magnetic ground state for each doping level x was determined by examining ferromagnetic (FM) and multiple antiferromagnetic (AFM) configurations. For monolayers, these include a checkerboard-type AFM ordering and three stripe-type patterns (Supplementary Fig. 2). Ground-state energies for all magnetic configurations were calculated using U_{eff} values varying between 0 and 4 eV, with atomic structures fully optimized under fixed spin configurations. The resulting energies (Supplementary Table 1) reveal a robust FM ground state for x > 0.5, where nonmagnetic (NM) and all AFM states remain energetically unfavorable across the tested U_{eff} range, consistent with prior studies [2]. Near intermediate doping ($0.375 \le x \le 0.5$), FM and certain AFM configurations exhibit comparable energies, with the phase boundary showing weak dependence on U_{eff}.

To assess the interlayer coupling in bulk Li_xFeSe and its impact on the magnetic phase diagram, interlayer FM and AFM couplings were evaluated alongside the in-plane magnetic configurations shown in Fig. S2. The relative stability of in-plane ordering patterns closely mirrors that of the monolayer, with the in-plane FM state most stable for $x > 0.5$. However, bulk calculations reveal weak AFM interlayer coupling, with an interaction strength of 1.1 meV/f.u. for LiFeSe (Ueff = 3 eV). Additional calculations using the strongly constrained and appropriately normed (SCAN) meta-GGA functional [3], as well as PBE and SCAN with van der Waals corrections (PBE-D3 [4] and SCAN-rvv10 [1,5]), consistently predict weak AFM interlayer coupling, with interaction strengths of 1.0, 2.3, and 3.9 meV/f.u. for PBE-D3+U (Ueff = 3 eV), SCAN,

and SCAN-rvv10, respectively. These results seem to suggest an AFM ground state for bulk Li_xFeSe across all dopings, despite the strong FM intralayer coupling observed at higher dopings.

Recent experiments, however, report structural transitions from the FeSe phase to Fe_2Se_2 phases upon Li intercalation [6], characterized by a doubling of the unit cell along the *c*-axis due to (1/2 1/2) in-plane sliding between adjacent FeSe layers. Such sliding is known to influence interlayer quasi-bonding and magnetic coupling in two-dimensional materials [7]. To explore these effects, interlayer sliding in LiFeSe was modeled for both (1/2 ,0) and (1/2, 1/2) configurations. These calculations reveal an FM ground state lower in energy than the AFM-A configuration by approximately 0.4 and 1.2 meV/f.u., respectively (with U_{eff}=3 eV), indicating FM interlayer coupling in the sliding-distorted structures. It is plausible that, in the realistic system, ferromagnetic FeSe layers at high doping levels are coupled ferromagnetically in some regions and antiferromagnetically in others, depending on local sliding distortions. This could result in weak net ferromagnetism. Notably, recent neutron scattering experiments have reported FM interlayer correlations even in pristine FeSe [8], suggesting the presence of intrinsic FM interlayer coupling in FeSe that may not be fully captured within the DFT framework. Together, these experimental and computational findings suggest weak FM interlayer interactions in Li_xFeSe. As a result, the monolayer phase diagram is adopted in the main text as a representative approximation of the bulk system.

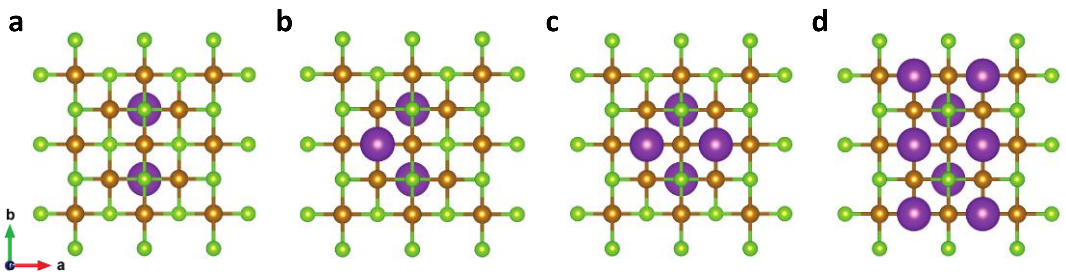

Supplementary Figure 1 Structural configurations of Li-intercalated FeSe. *Doping configurations for intermediate doping levels x=* ***a*** *1/4,* ***b*** *3/8,* ***c*** *1/2,* ***d*** *3/4 for a monolayer FeSe modeled with a 2 × 2 × 1 supercell. Bulk systems are constructed by stacking two such layers along the c-axis. Li, Fe, and Se ions are shown in purple, brown, and green, respectively.*

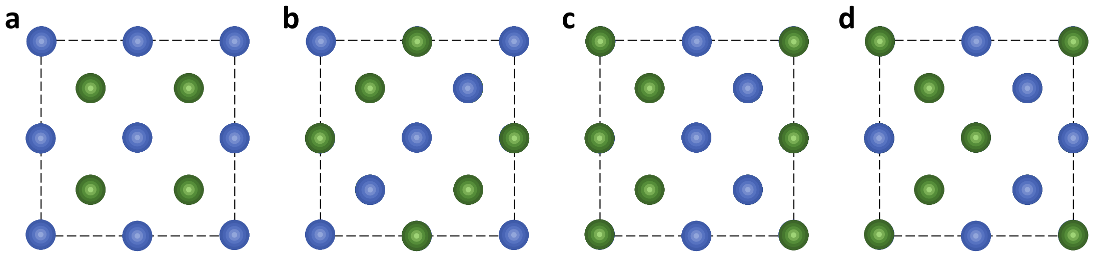

Supplementary Figure 2 **In-plane AFM configurations studied for Li-intercalated FeSe.** ***a-d*** *Four in-plane AFM spin orderings evaluated for* Li_xFeSe*:* ***a*** *checkerboard (AFM1),* ***b*** *collinear stripe (AFM2),* ***c*** *zigzag stripe type-1 (AFM3), and* ***d****, zigzag stripe type-2 (AFM4). Blue and green disks denote spin up and down, respectively. For clarity, only Fe sites are shown.*

Supplementary Table 1 DFT+*U* total energies of different magnetic configurations for monolayer Li_xFeSe. Total energies (in units of meV per formula unit, f.u.) for NM and different AFM states, calculated relative to the FM state.

Doping *x*	U_{eff} (eV)	NM	AFM-1	AFM-2	AFM-3	AFM-4
	0	528.3	486.4	370.0	211.4	306.4
	1	942.0	664.6	523.4	281.9	463.1
x=1	2	1379.9	866.9	657.0	327.0	356.6
	3	1845.0	964.1	410.9	307.0	299.5
	4	2335.5	361.1	896.4	412.3	208.8
	0	384.6	341.7	215.5	123.4	190.6
	1	799.8	470.5	327.0	184.5	244.9
x=3/4	2	1242.4	501.5	296.1	211.5	236.5
	3	1710.9	279.0	221.1	225.4	156.5
	4	2202.5	156.6	85.3	183.4	90.6
	0	168.0	79.5	42.2	80.9	33.1
	1	554.1	134.9	122.8	16.2	60.9
x=1/2	2	1012.9	113.6	93.0	43.4	45.8
	3	1506.3	176.4	26.9	-34.5	-46.7
	4	2133.5	315.6	19.8	-24.9	-41.9
	0	68.4	18.5	-38.9	-56.6	-42.5
	1	65.9	218.4	43.9	-20.7	0.1
x=3/8	2	868.5	109.0	-32.4	-63.3	-68.4
	3	1496.3	110.0	-16.9	-22.5	16.0
	4	2118.3	119.2	-56.1	-54.4	-36.0
	0	55.1	-0.1	-34.3	-31.6	-35.0
	1	323.3	42.5	-95.9	-110.3	-122.8
x=1/4	2	804.9	88.6	-104.4	-86.8	-70.0
	3	1387.5	-10.6	-122.0	-64.1	-119.8
	4	2073.3	-23.1	-95.0	-83.6	-88.8

Supplementary Note 2 Characterization of pristine FeSe

After the crystal growth, we carry out structural and physical property characterizations. Supplementary Figure 3a presents the X-ray diffraction (XRD) analysis. All four diffraction peaks are from FeSe. By using the higher order peaks, we estimate the *c*-axis lattice parameter to be about 5.525 Å, in agreement with previous reports on the β-phase FeSe [9]. Supplementary Figure 3b shows the temperature dependent resistance of the crystal. The ratio of room-temperature resistance to residual resistance (RRR) is 7.2. The superconducting onset temperature is 8 K (indicated by the black arrow in the inset) and the structural transition temperature is around 82 K (marked by the red arrow). These temperature values are consistent with former studies [10]. Supplementary Figure 3c shows the dc susceptibility data. It also confirms that the superconducting transition is at around 8 K. The dc susceptibility with ZFC also indicates bulk superconductivity with a full superconducting volume.

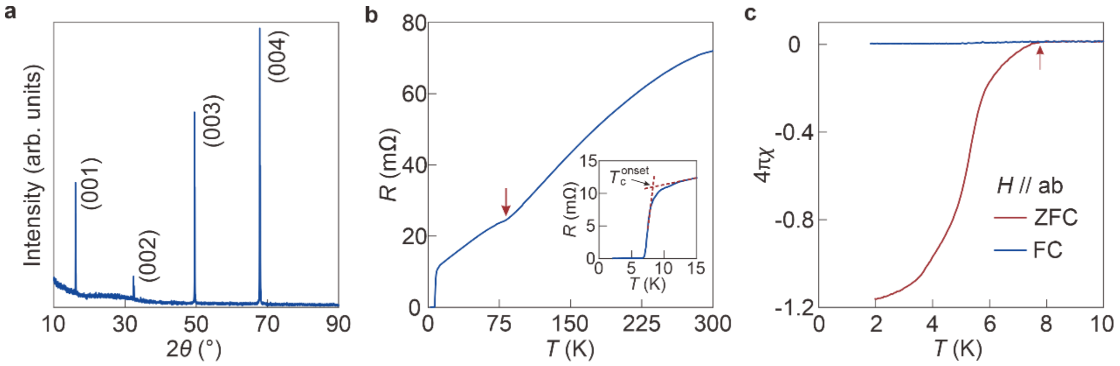

Supplementary Figure 3 a *X-ray diffraction data of the FeSe single crystal with Cu* K_α *radiation (λ = 1.5418 Å).* ***b*** *Temperature dependent resistance of FeSe. Red arrow indicates the temperature for the structural transition. Inset shows the data around the superconducting transition.* ***c*** *Temperature dependence of dc susceptibility at 10 Oe. The field is aligned to the a-b plane of FeSe. Red arrow indicates the superconducting transition temperature.*

Supplementary Note 3 Extended data of sample S1

We analyze the lithium content by examining the change in Hall densities of sample S1 (15 nm thick) before and after intercalation. At 50 K, the sample is tuned from being hole-dominant with $p_{2D} = 2.82 \times 10^{15}\ cm^{-2}$ to being electron-dominant with $n_{2D} = 6.29 \times 10^{15}\ cm^{-2}$ (Fig. 1j, m in the main text). The lithium content is estimated to be about 48%, assuming complete charge transfer from Li to the FeSe layers.

Supplementary Figure 4a and b collect the Hall resistance data from two pairs of contacts at 50 K for intercalated states #1 and #3, respectively. The longitudinal separation between each contact pair is 8 µm.

Supplementary Figure 5 shows the Hall resistance data for the gated state #1 at various temperatures. The hysteretic behavior persists to a temperature as high as 200 K (Supplementary Fig. 5a). We further show the Hall data obtained at 225 K in Supplementary Fig. 5b. Here, traces obtained from two opposite sweeps are non-overlapping, because lithium ions become mobile and the carrier density in the sample is changing during the sweeps. Despite the possible variation in the doping level, there exist clear jumps in the Hall resistances at the expected fields for the coercive fields (marked by vertical arrows). It indicates that ferromagnetism is still present at 225 K.

In Supplementary Fig. 6a, we further summarize the coercive field as a function of temperature for intercalated states #1 and #3. Supplementary Fig. 6b plots the anomalous Hall resistance (R_{AH}) as a function of the corresponding longitudinal resistance in the temperature window from 150 to 50 K. The linear behavior indicates that skew scattering is the dominant origin for the anomalous Hall effect.

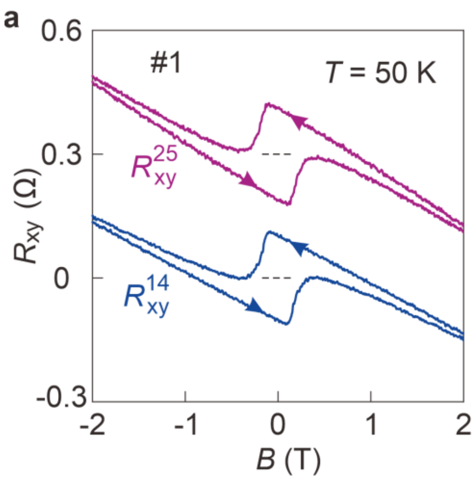

Supplementary Figure 4 a *and* ***b*** *Hall resistances as a function of perpendicular magnetic field from two pairs of contacts on the sample S1 for intercalated states #1 (a) and #3 (b) at 50 K. Curves are vertically offset for clarity. Dashed lines mark zero Hall resistances for different data sets. Arrows indicate the sweeping directions of the magnetic field.*

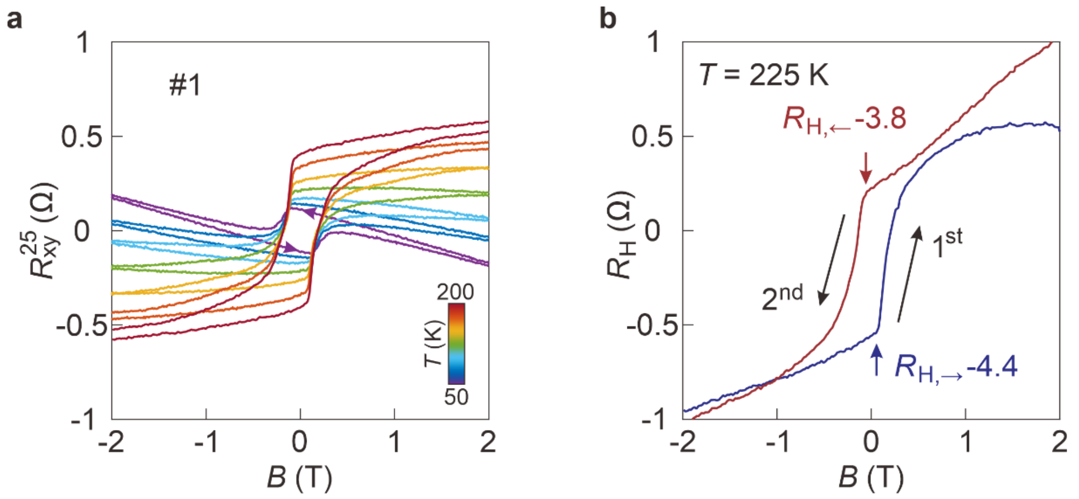

Supplementary Figure 5 a *and* ***b*** *Hall resistances at a set of temperature points after lithium intercalation. The temperature increases in a step of 25 K from 50 K to 200K in* ***a****. Curves are vertically offset in* ***b****.*

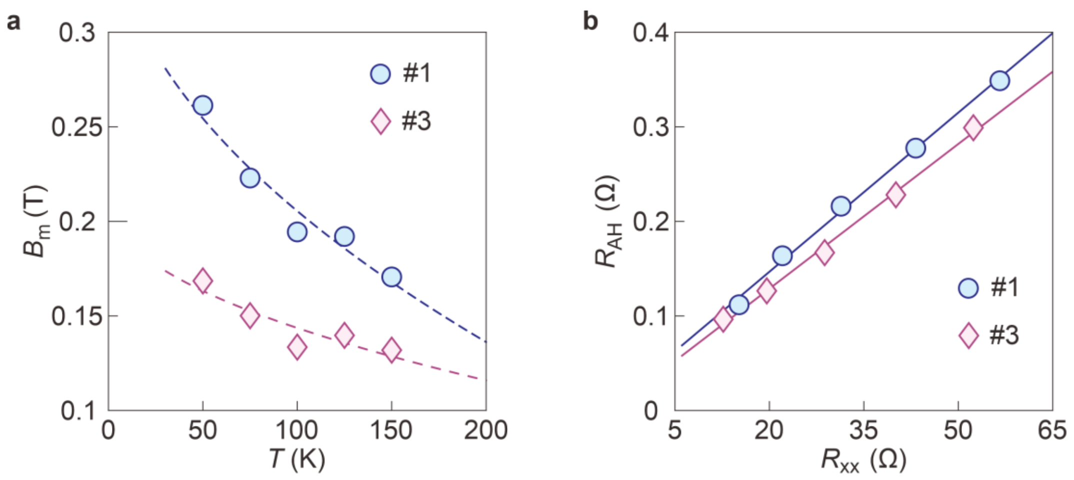

Supplementary Figure 6 a *Temperature dependence of the coercive field for gated state #1 (circles) and #3 (diamonds).* ***b*** *Anomalous Hall resistance as a function of longitudinal resistance, with the solid line representing a linear fit to the data.*

Supplementary Note 4 Extended data of sample S2

Supplementary Figure 7 shows data collected from sample S2 (14 nm thick). Supplementary Figure 7a and b compares the transport data of sample S2 before and after lithium intercalation. Sample S2 in the pristine state shows a standard linear response in the Hall resistance, suggesting the absence of ferromagnetic ordering. Figures 7c and 5d show Hall resistances measured from three pairs of contacts, with a longitudinal separation of 10 μm between each pair. All measurements exhibit the same hysteresis loop, indicating mesoscopic homogeneity.

Supplementary Figure 7e collects the Hall resistance data after lithium intercalation at various temperatures. The anomalous Hall signal becomes more prominent at higher temperature while the corresponding coercive field becomes smaller (further summarized in Supplementary Fig. 7f). At 180 K, the two traces for the two opposite directions are not overlapping at high magnetic fields. This is because lithium ions already become mobile and the carrier density starts to vary. Still, we observe clear jumps at the coercive fields, indicating the persistence of ferromagnetism.

Supplementary Figure 8a and b plot the Hall resistance data from pair 1-4 of S2. At small magnetic fields, there exist notable fluctuations at temperatures below 45 K. This may stem from inhomogeneity. We note that the region around contact 4 may have a different doping, as reflected by the broader superconducting transition from pair 4-5. It is further confirmed by the n_H extracted from pair 1-4 and 2-5 (Supplementary Fig. 8c). Despite the fluctuations in small magnetic fields, the general temperature evolution seen from this pair of contacts is consistent with that measured from the other pair 2-5 shown in the main text. This is further illustrated in Supplementary Fig. 8c, in which we compare R_{AH}/n_H extracted from the two pairs of contacts. We note that the larger n_H measured from pair 1-4 reflects the thicker region, which can be also identified from the optical image (inset of Supplementary Fig. 7a)

Supplementary Figure 9 plots the extracted R_{AH} as a function of the corresponding

longitudinal resistance at zero field. The linear scaling indicates the presence of skew scattering.

In Supplementary Fig. 10a, we compare the magneto-resistance measured at 42 K but with three different rates: 50 mT/min, 5 mT/min and 2.5 mT/min. In Supplementary Fig. 10b, we compare the data obtained with two different rates 5 mT/min and 2.5 mT/min. In Supplementary Fig. 10c, we plot the loop area of the hysteresis (A_{MR}) as a function of the sweeping rate. The area saturates at the low sweeping rate. We therefore conclude that the hysteresis is not caused by magnetocaloric effect. Also, we use 5 mT/min as the rate for obtaining the data in Fig. 2b, c because it is already a sufficiently low sweeping rate.

Supplementary Figure 11a shows the magnetic field dependences of longitudinal resistances in the normal state (50 K) at several selected tilting angles. They are measured simultaneously with the Hall resistance data shown in Fig. 3a. In Supplementary Fig. 11b, we summarize the angular dependence of the coercive fields (indicated by the arrows in Supplementary Fig. 11a). With decreasing θ, the coercive field becomes substantially larger. Supplementary Figure 11c collects the Hall coefficient estimated from the high field section of data in Fig. 3a in the main text. The sin(θ) dependence of Hall coefficient indicates the two-dimensional transport behavior of FeSe flakes.

Supplementary Figures 12 and 13 present the temperature dependent resistances from two pairs of contacts at a set of magnetic fields under different tilted angles. In contrast to the standard longitudinal resistance probed by the pair of contacts 1 and 2, the pair 2-5 aligns transversely to the current flow. Still, due to the slight misalignment, longitudinal resistance mixes into the measured resistance. The contribution from the Hall effect becomes prominent once a perpendicular magnetic field is applied. It gives rise to the general decrease of resistance at higher magnetic fields in Supplementary Fig. 13a-c. When the magnetic field is applied in the plane

(Supplementary Fig. 13d), however, no Hall effect is generated and the measured resistance traces show clearly the shift to higher temperatures with increasing fields. This shifting to higher temperatures is more pronounced than those presented in Fig. 3c, d, presumably because pair 2-5 probes a smaller area with even higher uniformity.

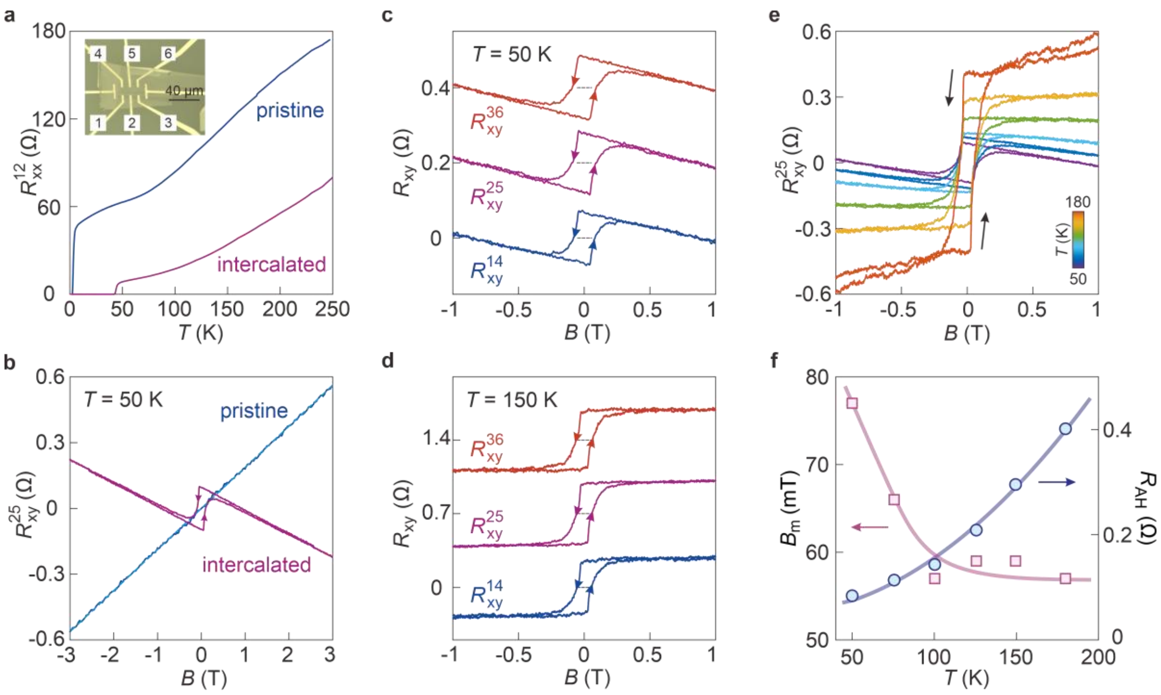

***Supplementary Figure 7 a, b** Temperature dependent resistances and Hall resistances at 50 K for sample S2 (optical image shown in the inset) before and after lithium intercalation. The dashed line (light blue) in **b** is a linear fit to the data of pristine state. **c** and **d** Hall resistances as a function of perpendicular magnetic field from three pairs of contacts. **e** Hall resistances of S2 after lithium intercalation. The temperatures are 50, 75, 100, 125, 150 and 180 K. **f** Temperature dependence of* R_{AH} *(circles) and the coercive field (squares).*

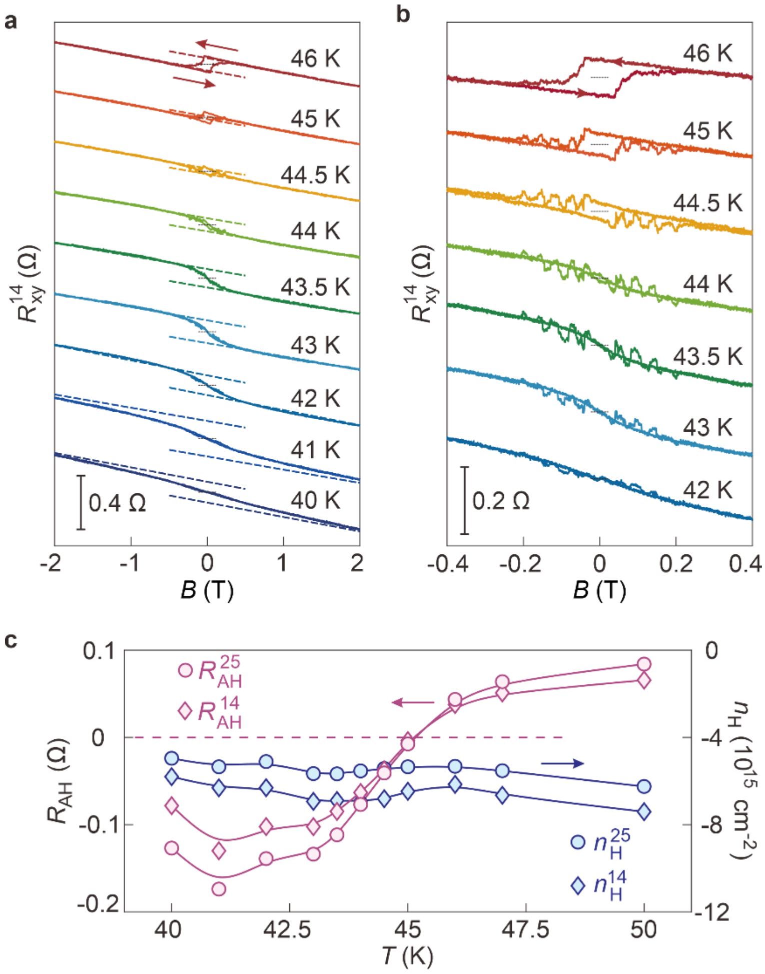

Supplementary Figure 8 a, b *Hall resistance as a function of perpendicular magnetic field measured at a set of temperature points from the contacts 1 and 4 on sample S2(indicated in the inset of Fig. 1b). Curves are vertically offset for clarity. Dotted lines indicate zero Hall resistances for different data sets. Dashed lines in* ***a*** *represent linear fits to the data at high fields.* ***c*** *Temperature dependence of* R_{AH} *and* n_H *from pair 1-4 (diamonds) and pair 2-5 (circles).*

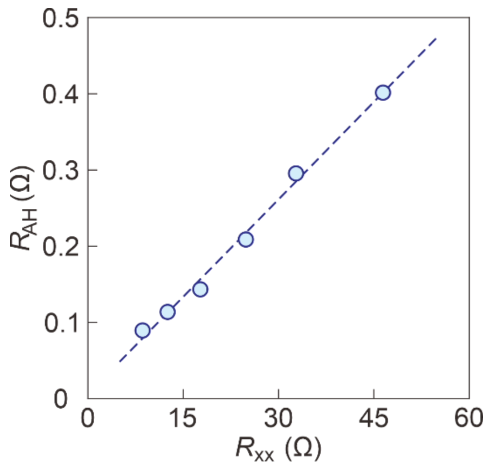

Supplementary Figure 9 *Anomalous Hall resistance as a function of longitudinal resistance. The dashed line is a linear fit to the data.*

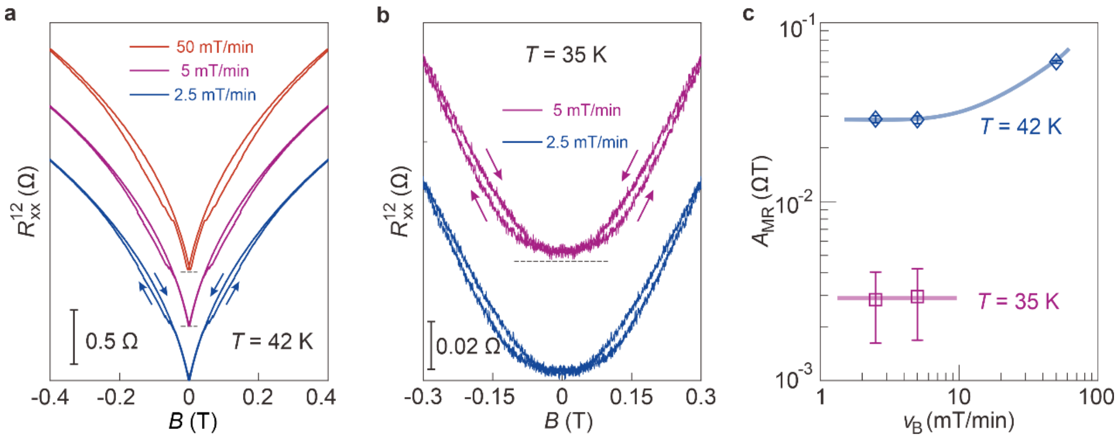

Supplementary Figure 10 a, b *Longitudinal resistance of sample S2 as a function of perpendicular magnetic field measured at different sweeping rates. Curves are vertically offset for clarity. Dashed lines indicate zero longitudinal resistances.* ***c*** A_{MR} *as a function of the* sweeping rate at 42 K (diamonds) and 35 K (squares). Error bars are estimated from the standard deviation of the resistances measured at high magnetic fields.

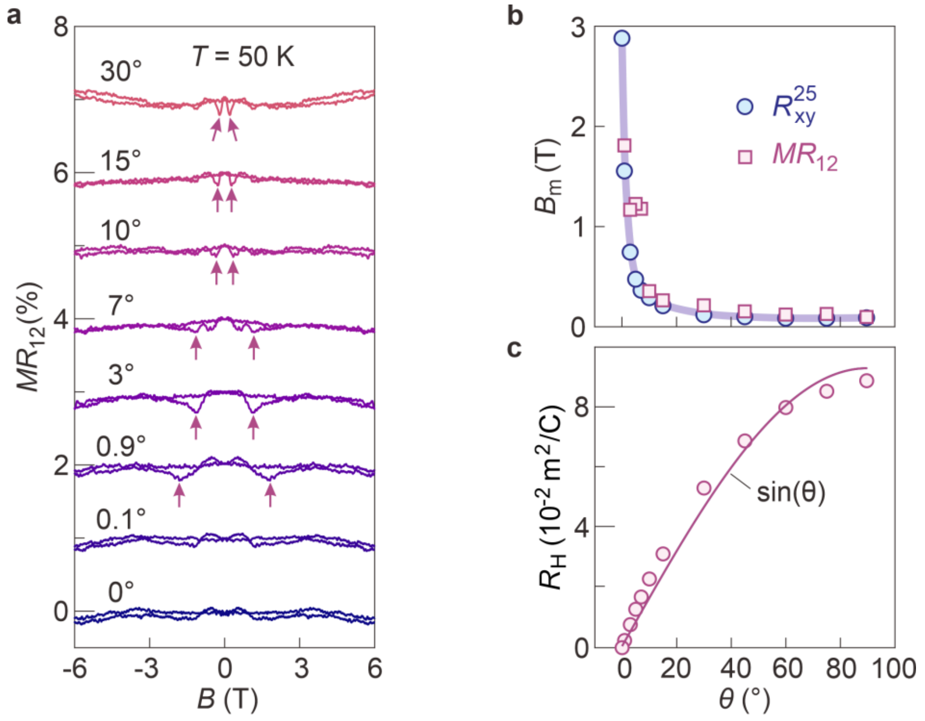

Supplementary Figure 11 a *Magnetoresistance [MR=*$\left(R_{xx}(B) - R_{xx}(0)\right)/R_{xx}(0)$*] at selected tilting angles. Arrows mark the local minima in the curves. Their magnetic field positions are taken as the coercive fields.* ***b*** *Coercive fields from Hall and longitudinal resistances as a function of tilting angle.* ***c*** *Angular dependence of the ordinary Hall coefficients obtained from the high field section of Hall resistance: [4, 8] T. Solid curve is a fit by using the* $sin(\theta)$ *dependence.*

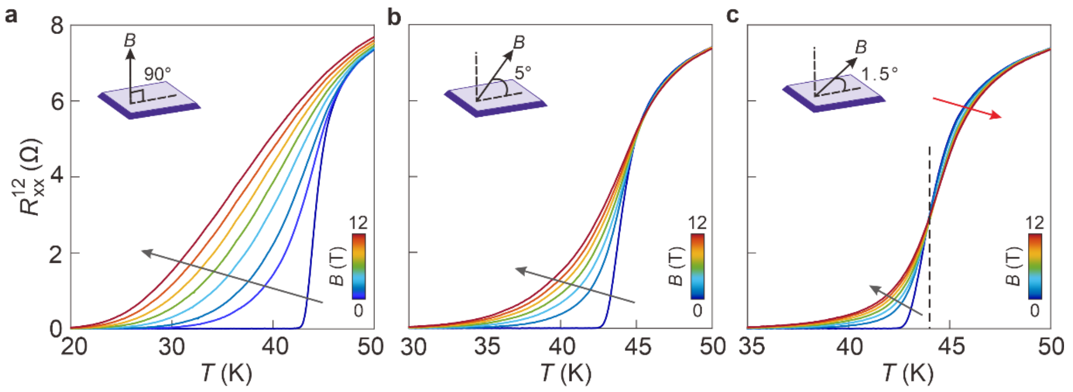

Supplementary Figure 12 a-c *Temperature dependent resistances at a set of magnetic fields* under different tilting angles (*shown in the inset*) *from the contacts 1 and 2 on sample S2. Gray and red arrows indicate respectively the conventional and anomalous field responses.*

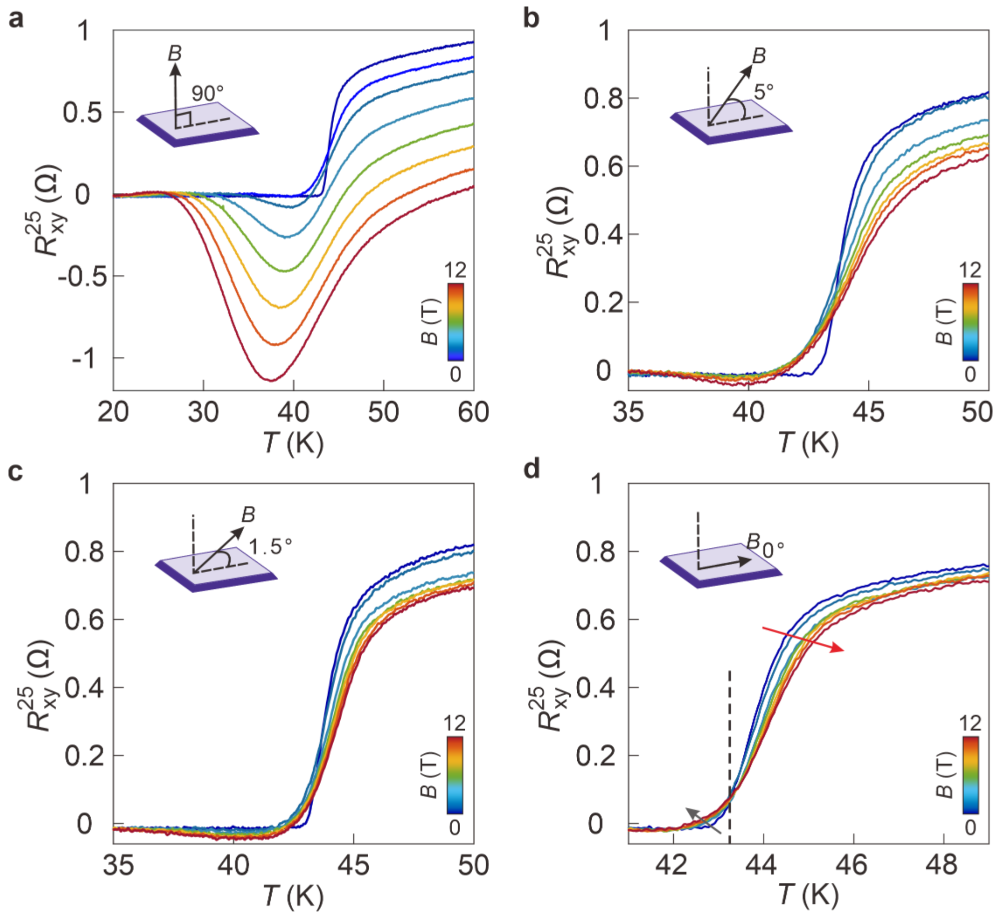

Supplementary Figure 13 a-d *Temperature dependent resistances at a set of magnetic fields* under different tilting (*shown in the inset*) *from the contacts 2 and 5 on sample S2. Gray and red arrows indicate respectively the conventional and anomalous field responses.*

Supplementary Note 5 Broadening of superconducting transition

We quantitatively analyze the broadening of the superconducting transition in sample S2. We define the superconducting transition width ΔT as the temperature window where the resistance varies from 10% to 90% of the normal state value. The broadening induced by the magnetic field is calculated as $\Delta T - \Delta T_{B=0}$. We then evaluate the corresponding number of vortices that penetrate through the sample by using:

$$N = \frac{BSsin\theta + Bwdcos\theta}{\Phi_0},$$

where $\Phi_0 = h/2e$ is magnetic flux quantum, $w/d/S$ is the width/thickness/area of sample S2 and θ is the tilting angle, as shown in Supplementary Fig. 14a. The formula above takes into account both the Abrikosov vortices formed by the perpendicular magnetic field and the Josephson vortices by the in-plane magnetic field. Supplementary Fig. 14b plots the broadening for data at different tilting angles as a function of N. Notably, these data points fall on the same trend. Although the data points at the nominal tilting angle of $0°$ shows obvious discrepancy, they immediately fall on the trend described by the data at other tilting angles if we assume a slightly misaligned angle of $0.2°$ (black arrow). This slight misalignment is likely caused by the slight bending of the sample. The general trend followed by data points collected from different tilting angles clearly indicates that vortices play a dominant role in broadening the superconducting transition.

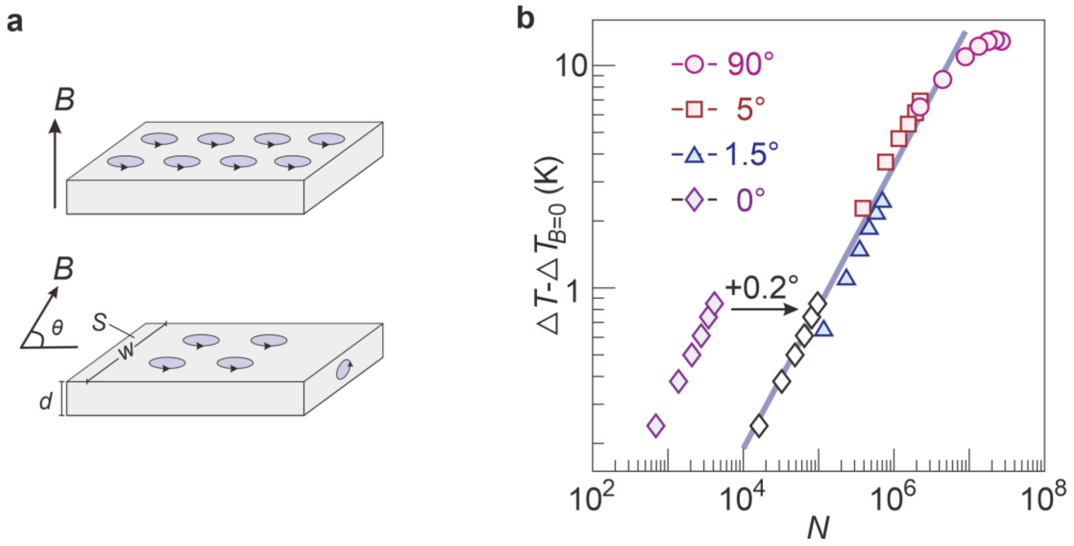

Supplementary Figure 14 a *Schematic drawing of vortices at different tilting angles.* ***b*** *Broadening of the superconducting transition at different tilting angles as a function of the number of vortices going through the sample.*

Supplementary Note 6 Magnetometry after de-intercalation

Supplementary Figure 15 exhibits the magnetometry of the sample S3 after de-intercalation process. The magnetometry image without any ferromagnetic pattern indicates that the sample returns to a nonmagnetic state after the withdrawal of lithium ions.

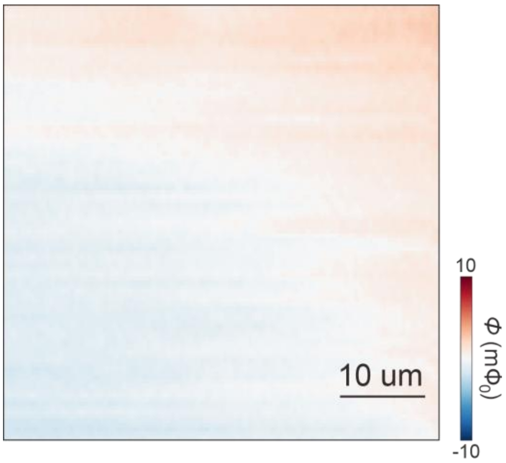

Supplementary Figure 15 *Magnetometry image* of the sample S3 *measured at 16 K* after de-intercalation process.

Supplementary Note 7 Extended data of sample S5

In the main text, we show data from samples S1-S4. Here, we present data from sample S5 under various gating conditions. Based on its optical contrast, we estimate that sample S5 has a thickness of about 10 nm (thinner than S2). We use #0 to denote the pristine state. We apply positive backgate voltages at 300 K for the gated state #1 to #3 (#1: 2 V to 2.8 V for 25 min, #2: 2.8 V for 5min, #3: 2 V to 2.7 V for 7 min) and apply negative voltages (-0.5 V to -0.8 V for 10 min at 300 K) for the state #4. Supplementary Figure 16a shows the temperature dependent resistances of the five different states. State #1 shows slightly enhanced superconductivity in comparison to state #0, indicating a small amount of intercalated lithium ions. Interestingly, this state shows a linear Hall trace without any indication of ferromagnetism (Supplementary Fig. 16b). Further intercalation results in states #2 and #3 with the superconducting onset being promoted to around 40 K. These two states show clear jumps in the Hall resistances, suggesting the emergence of ferromagnetism (Supplementary Fig. 16c, d). For state #4, we withdraw lithium ions from the sample such that it goes back to the low-T_{sc} phase. As shown in Supplementary Fig. 16b, the jump in Hall resistances disappear and the Hall resistance recovers the linear behavior. This study clearly indicates that ferromagnetism correlates with the high-T_{sc} phase induced by lithium intercalation.

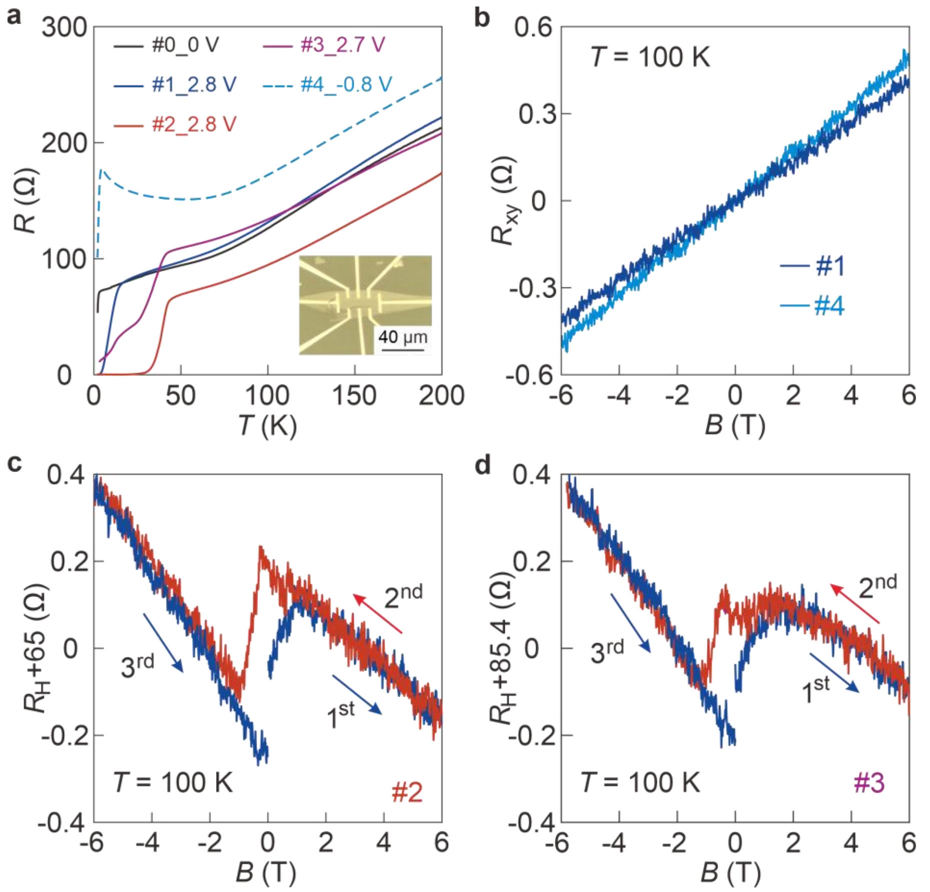

***Supplementary Figure 16 a** Temperature dependent resistances for sample S5 (optical image shown in the inset). Numbers mark the consecutive gating sequence. Solid/dashed lines represent the intercalated/de-intercalated state. **b-d** Hall resistances measured at 100 K for different states. Data in **b** are anti-symmetrized from the raw data.*

Supplementary References:

[1] Vydrov, O. A., Voorhis, T. V. Nonlocal van der Waals density functional: The simpler the Better. *J. Chem. Phys.* **133**, 244103 (2010).

[2] Li, Y., Li, J., Li, Y., Ye, M., Zheng, F., Zhang, Z., Fu, J., Duan, W., Xu, Y. High-Temperature Quantum Anomalous Hall Insulators in Lithium-Decorated Iron-Based Superconductor Materials. *Phys. Rev. Lett.* **125**, 086401 (2020).

[3] Sun, J., Ruzsinszky, A., Perdew, J. P. Strongly Constrained and Appropriately Normed Semilocal Density Functional. *Phys. Rev. Lett.* **115**, 036402 (2015).

[4] Grimme, S., Antony, J., Ehrlich, S., Krieg, H. A consistent and accurate ab initio parametrization of density functional dispersion correction (DFT-D) for the 94 elements H-Pu. *J. Chem. Phys.* **132**, 154104 (2010).

[5] Sabatini, R., Gorni, T., de Gironcoli, S. Nonlocal van der Waals density functional made simple and efficient. *Phys. Rev. B* **87**, 041108 (2013).

[6] Lei, B., Wang, N. Z., Shang, C., Meng, F. B., Ma, L. K., Luo, X. G., Wu, T., Sun, Z., Wang, Y., Jiang, Z., Mao, B. H., Liu, Z., Yu, Y. J., Zhang, Y. B., Chen, X. H. Tuning phase transitions in FeSe thin flakes by field-effect transistor with solid ion conductor as the gate dielectric. *Phys. Rev. B* **95**, 020503 (2017).

[7] Jiang, P., Wang, C., Chen, D., Zhong, Z., Yuan, Z., Lu, Z. Y., Ji, W. Stacking tunable interlayer magnetism in bilayer CrI_3. *Phys. Rev. B* **99**, 144401 (2019)

[8] Ma, M., Bourges, P., Sidis, Y., Sun, J., Wang, G., Iida, K., Kamazawa, K., Park, J. T., Bourdarot, F., Ren, Z., Li, Y. Ferromagnetic interlayer coupling in $FeSe_{1-x}S_x$ superconductors revealed by inelastic neutron scattering. *Phys. Rev. B* **110**, 174503 (2024)

[9] Pomjakushina, E., Conder, K., Pomjakushin, V., Bendele, M., Khasanov, R. Synthesis, crystal structure, and chemical stability of the superconductor $FeSe_{1-x}$. *Phys. Rev. B* **80**, 024517 (2009).

[10] Böhmer, A. E., Taufour, V., Straszheim, W. E., Wolf, T., Canfield, P. C. Variation of transition temperatures and residual resistivity ratio in vapor-grown FeSe. *Phys. Rev. B* **94**, 024526 (2016).
